# Sex difference in the age-related decline of global longitudinal strain of left ventricle

**DOI:** 10.1038/s41598-023-42286-9

**Published:** 2023-10-27

**Authors:** Kyung-Yeon Lee, Hack-Lyoung Kim, Kyung-Jin Kim

**Affiliations:** 1 Division of Cardiology, Department of Internal Medicine, Boramae Medical Center, Seoul National University College of Medicine, Seoul, Korea; 2 Department of Internal Medicine, Ewha Womans University Medical Center, Ewha Womans University School of Medicine, 1071 Anyangcheon-Ro, Yangcheon-Gu, Seoul, 07985 Korea

**Keywords:** Ultrasound, Heart failure, Risk factors

## Abstract

Global longitudinal strain (GLS) is a valuable indicator of subclinical myocardial dysfunction. Whether the effect of aging on subclinical left ventricular dysfunction is sex-specific is not well documented. This study aimed to identify age-related changes in GLS according to sex in patients with a normal left ventricular ejection fraction (LVEF). In this cross-sectional, single-center cohort study in Korea, participants who underwent GLS measurement using 2D speckle-tracking echocardiography were retrospectively reviewed, and participants with normal LVEF (≥ 55%) without documented cardiovascular disease were included. Reduced GLS was defined as absolute values below 18%. Of 682 study participants (mean age, 58; female, 51.5%), 209 (30.6%) had reduced GLS. Females with reduced GLS were older than those with normal GLS (68 vs. 58 years, *P* < 0.001); with no difference of age in males (55 vs. 57 years; *P* = 0.265). Univariate analysis showed age to correlate significantly with reduced GLS only in female (*r* =  − 0.364; *P* < 0.001). In multivariable analysis, female > 66 years old had significantly higher risk of reduced GLS (Odds ratio 2.66; 95% CI 1.22–5.76; *P* = 0.014). In participants with normal LVEF, GLS decreased with age in females but not in males. Particularly, females aged 66 years and older had a significantly higher risk of reduced GLS. These findings suggest that GLS could be a valuable parameter for assessing subclinical cardiac dysfunction, especially in older females.

## Introduction

Heart failure with preserved ejection fraction (HFpEF) is one of the major challenges in recent cardiology, given its increasing prevalence and high morbidity and mortality^1^. However, relative to the clinical importance of HFpEF, proven therapy is limited and diagnosis is challenging^2^. The pathophysiological diversity of HFpEF may contribute to these limitations; however, despite the diversity, one consistent observation is that HFpEF is prevalent in elderly females^1^. Yet, the mechanisms underlying the higher prevalence of HFpEF in elderly females remains to be resolved^3^.

Recently, reduced longitudinal systolic function has been suggested as an important change in HFpEF^4^. This change has also been observed with aging and in females as well. Aging is associated with left ventricular (LV) remodeling process, which increases myocardial twist and decreases longitudinal function^5^. Sex also has a significant impact on myocardial remodeling^5,6^, and remodeling was more prominent in females than in males^6^ which could in turn reduce longitudinal function. Therefore, exploring different effects with aging by sex may improve our understanding of HFpEF.

Global longitudinal strain (GLS) is the echocardiographic technique that measures myocardial mechanics by tracking myocardial muscle movement^7,8^. This speckle tracking technique was proposed to reflect early changes in LV longitudinal function when the LV ejection fraction (LVEF) remains normal^9–11^. A meta-analysis reported that LV longitudinal systolic function measured using GLS was significantly lower in patients with HFpEF^12^. As expected, GLS reduces with age^13^. However, regarding sex, the result is inconsistent in that GLS is reported to be worse in males than females with normal LVEF^10,14^. This contradictory result raises doubts about whether GLS reduction with aging differ by sex. If so, it might provide understanding of the mechanism of HFpEF by which longitudinal systolic function decreases with aging differently by sex.

Therefore, this study sought to evaluate sex differences in LV GLS with normal LVEF, specifically with respect to aging.

## Methods

### Study participants

The medical records of patients who underwent transthoracic echocardiography for GLS measurement and who had no documented cardiovascular disease were reviewed retrospectively. A total of 1,100 patients were initially included, and 418 patients were excluded according to the following exclusion criteria: the presence of reduced LVEF (< 55%), history of myocardial infarction or coronary artery disease, cardiac arrhythmia, valvular dysfunction greater than a mild degree, pericardial effusion, congenital heart disease, or extra-cardiac diseases (i.e., end-stage renal failure, chronic lung disease, liver cirrhosis, malignancy). Finally, 682 patients were included in this study. This study was conducted in accordance with the 2013 revision of the Helsinki Declaration. This study was approved by the Institutional Review Board (IRB) of the Seoul Metropolitan Government-Seoul National University Boramae Medical Center (IRB No. 20-2023-12). Owing to the retrospective study design and routine nature of the data obtained, the requirement for informed consent was waived by the IRB of the Seoul Metropolitan Government-Seoul National University Boramae Medical Center.

### Clinical data collection

A prior diagnosis of hypertension, current use of antihypertensive drugs, or systolic and/or diastolic blood pressure over 140/90 mmHg was defined as hypertension. A prior diagnosis of diabetes mellitus, current use of anti-diabetic drugs, or a fasting blood glucose level above 126 mg/dL was defined as diabetes mellitus. A prior diagnosis of dyslipidemia, current use of anti-dyslipidemic drugs, or low-density lipoprotein cholesterol level $\ge$ 160 mg/dL was defined as dyslipidemia. Obesity was defined as a body mass index $\ge$ 25 kg/m^2^^15^.

### Transthoracic echocardiography

Echocardiographic images were acquired and measured according to the recommendations of the American Society of Echocardiography and European Association of Cardiovascular Imaging^16^. All transthoracic echocardiography and speckle tracking strain imaging were performed using the same ultrasound machine (GE Medical Systems, Milwaukee, WI, USA).

LV end-diastolic dimension, LV end-systolic dimension, and wall thickness were measured using M-mode, and M-mode echocardiography was conducted on parasternal views. LV mass index was calculated using a validated linear method and indexed to the body surface area. LVEF was measured using the Simpson’s biplane method on apical 4-chamber and 2-chamber views.

### Strain echocardiography

Speckle-tracking analysis was conducted during echocardiographic examinations by two experienced sonographers. All echocardiographic parameters were averaged over 3 cardiac cycles. The LV endocardial border was automatically traced using commercially available software (GE Medical Systems) and was manually adjusted by an experienced sonographer. GLS value was measured from the average of the apical 4-chamber, 2-chamber, and 3-chamber views.

We took the absolute value |x| for a simpler interpretation given the negative GLS values; hence, we described a more positive value as “reduced”. Reduced GLS was defined as less than 18%, and normal GLS was defined as more than 18%^11,17,18^.

### Statistical analysis

All statistical analyses were performed using SPSS statistics version 26.0 (IBM Corp., Armonk, NY, USA). Continuous variables are presented as means and standard deviations, and categorical variables are presented as frequencies and percentages. Comparisons between the two groups were conducted using Student’s t-test for continuous variables and the Chi-square test for categorical variables. Univariate and multivariate binary logistic regression analyses were used to identify significant factors correlated with reduced GLS. Receiver operating characteristic (ROC) curve analysis was used to assess the correlation between sex and reduced GLS and to identify the optimal cutoff value of age for predicting reduced GLS risk. The choice of cutoff value was made using the concordance probability method. A comparison of the area under the curve (AUC) of reduced GLS between sexes was made to assess the influence of age on reduced GLS. Statistical significance was set at *P* < 0.05.

## Results

### Baseline characteristics of total study participants

Of the 682 participants of this study, 209 (30.6%) had reduced GLS (Table 1). Participants with reduced GLS were predominantly males. Age did not differ according to GLS. Hypertension, diabetes, and obesity were more prevalent in participants with reduced GLS. Cholesterol levels and dyslipidemia were not significantly different between the normal and reduced GLS groups of participants, but serum triglyceride levels were higher in the reduced GLS group. The reduced GLS group took significantly more calcium channel blockers, renin-angiotensin blockers, and diuretics than the normal GLS group.

**Table 1:** Clinical characteristics and echocardiographic findings of study subjects.

	Total (n = 682)	Reduced GLS (< 18%) (n = 209)	Normal GLS ($\ge$ 18%) (n = 473)	*P*
Age, years	58.2 ± 13.2	59.3 ± 14.4	57.6 ± 12.6	0.128
Men, sex	331 (48.5)	143 (68.4)	188 (39.7)	< 0.001
Women, sex	351 (51.5)	66 (31.6)	285 (60.3)	< 0.001
Body mass index, kg/m2	24.5 ± 2.93	25.2 ± 3.13	24.3 ± 2.80	< 0.001
SBP	130.5 ± 17.7	135.9 ± 19.4	127.9 ± 16.2	< 0.001
DBP	79.1 ± 11.9	83.2 ± 11.9	77.1 ± 11.3	< 0.001
HR	67.8 ± 10.7	69.9 ± 11.9	66.8 ± 10.0	0.007
Alcohol	183 (26.8)	74 (35.4)	109 (23.0)	0.001
Cardiovascular risk factors				
Hypertension	339 (49.7)	126 (60.3)	213 (45.0)	< 0.001
Diabetes mellitus	106 (15.5)	42 (20.1)	64 (13.5)	0.029
Dyslipidemia	132 (19.4)	47 (22.5)	85 (18.0)	0.169
Obesity	284 (41.6)	105 (50.2)	179 (37.8)	0.002
Current smoking	119 (17.4)	58 (27.8)	61 (12.9)	< 0.001
Lab findings				
Glucose, mg/dL	109.2 ± 29.0	113.6 ± 33.4	107.3 ± 26.6	0.009
Glycated hemoglobin, %	5.90 ± 0.72	6.01 ± 0.88	5.84 ± 0.63	0.012
GFR_MDRD	90.5 ± 21.2	88.2 ± 24.3	91.5 ± 19.6	0.059
Total cholesterol	183.2 ± 37.8	182.4 ± 39.6	183.5 ± 37.0	0.734
LDL cholesterol	114.4 ± 34.5	112.6 ± 36.4	115.2 ± 33.7	0.399
HDL cholesterol	50.6 ± 12.8	47.9 ± 12.0	51.8 ± 12.9	< 0.001
Triglyceride	118.6 ± 66.6	131.4 ± 73.7	113.0 ± 62.5	0.001
C-reactive protein	0.38 ± 1.26	0.44 ± 1.43	0.35 ± 1.17	0.468
Concomitant medications				
Calcium channel blocker	211 (30.9)	78 (37.3)	133 (28.1)	0.017
Beta-blocker	106 (15.5)	41 (19.6)	65 (13.7)	0.051
Renin-angiotensin system blocker	186 (27.3)	77 (36.8)	109 (23.0)	< 0.001
Diuretic	34 (5.0)	16 (7.7)	18 (3.8)	0.033
Statin	278 (40.8)	95 (45.5)	183 (38.7)	0.097
Echocardiographic parameter				
GLS, %	19.1 ± 2.35	16.4 ± 1.35	20.2 ± 1.62	< 0.001
LV ejection fraction, %	67.0 ± 4.39	65.6 ± 4.60	67.6 ± 4.14	< 0.001
LV end-diastolic dimension, mm	48.0 ± 3.48	48.6 ± 3.78	47.7 ± 3.31	0.004
LV end-systolic dimension, mm	30.1 ± 3.04	31.0 ± 3.30	29.7 ± 2.82	< 0.001
LV septal wall thickness, mm	8.68 ± 1.19	9.15 ± 1.11	8.48 ± 1.16	< 0.001
Posterior wall thickness, mm	8.61 ± 1.14	9.06 ± 1.06	8.41 ± 1.11	< 0.001
LV mass index, g/m^2^	83.7 ± 17.3	88.9 ± 18.9	81.4 ± 16.0	< 0.001
E wave velocity, cm/s	0.66 ± 0.25	0.64 ± 0.39	0.67 ± 0.15	0.67
A wave velocity, cm/s	0.74 ± 0.39	0.77 ± 0.65	0.73 ± 0.19	0.157
E/A	0.96 ± 0.35	0.89 ± 0.32	0.99 ± 0.36	< 0.001
Deceleration time, ms	209.3 ± 46.6	209.3 ± 50.6	209.3 ± 44.8	0.996
Septal e' velocity	7.48 ± 3.61	6.59 ± 2.12	7.88 ± 4.04	< 0.001
E/e'	9.43 ± 3.07	9.90 ± 3.58	9.23 ± 2.79	0.016
LA volume index, mL/m^2^	28.5 ± 7.89	29.4 ± 9.37	28.1 ± 7.10	0.042
TR Vmax, m/s	2.24 ± 0.26	2.23 ± 0.28	2.25 ± 0.26	0.513

Numbers are mean ± standard deviation or n (%). GLS, global longitudinal strain.

HTN : previous diagnosis, current antihypertensive medications or SBP and/or DBP ≥ 140/90.

DM : previous diagnosis, current anti-diabetic medications or fasting blood glucose level ≥ 126 mg/dL.

DL : previous diagnosis, current use of anti-dyslipidemic medications or LDL ≥ 160 mg/d.

### Echocardiographic findings in study participants

In all participants, mean GLS values of the reduced and normal GLS groups were 16.4 ± 1.35% and 20.2 ± 1.62%, respectively. LVEF was lower in the reduced GLS group than normal GLS group (65.6% ± 4.60% vs. 67.6% ± 4.14%, *P* < 0.001). The reduced GLS group had a thicker LV wall, and higher LV mass index, and left atrial (LA) volume index than the normal GLS group. E/A ratio and septal E’ velocity were lower in the reduced GLS group than normal GLS group (Table 1).

### Age- and sex-related differences

**Figure 1:**
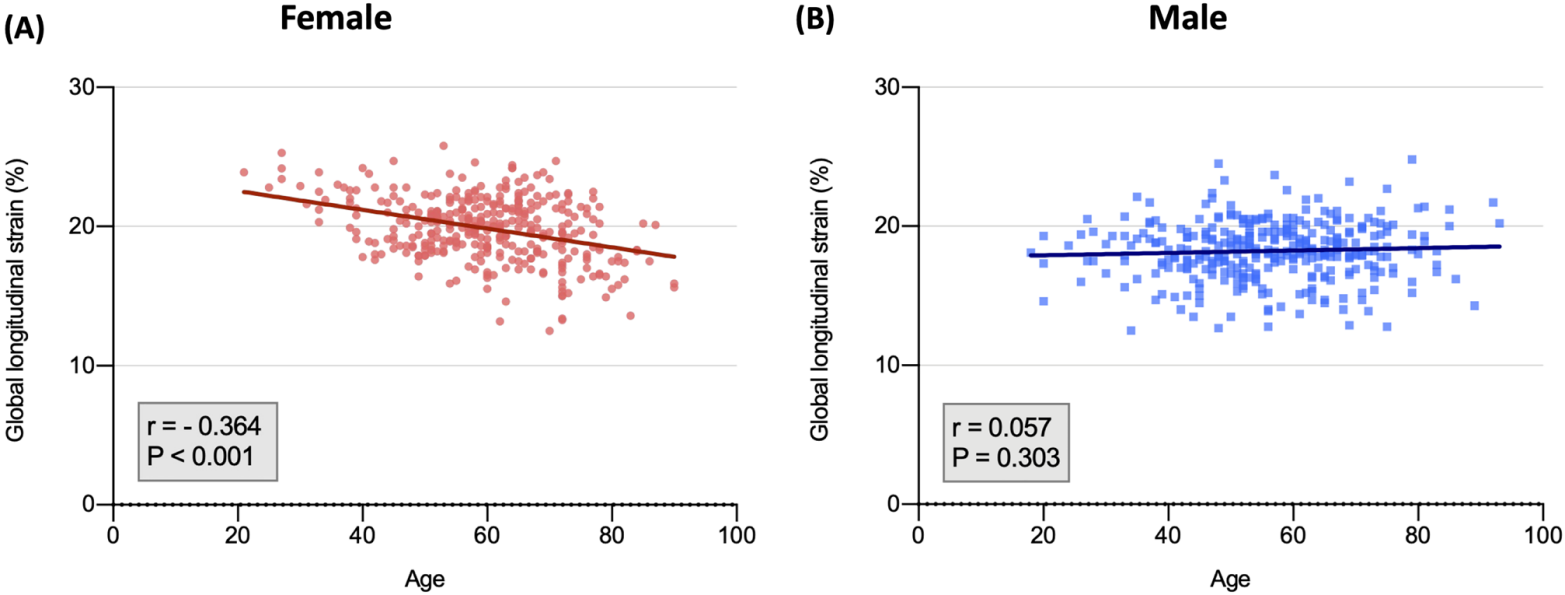
Correlation between age and global longitudinal strain (females, r =—0.364, *P* < 0.001; males, r = 0.057, *P* = 0.303).

Baseline characteristics of the study participants, with respect to sex are shown in Table 2. Increasing age was associated with reduced GLS, but this effect was observed only in females; age difference between the groups with reduced and normal GLS was significant in females (68 vs. 58 years; *P* < 0.001) but not males (55 vs. 57 years; *P* = 0.265). In univariate linear correlation analyses, age was negatively associated with GLS in females (*r* =  − 0.364; *P* < 0.001; Fig. 1A) but not males (*r* = 0.057; *P* = 0.303; Fig. 1B). The reduced GLS group had higher systolic and diastolic blood pressures in both males and females, but more female had hypertension. Participants with reduced GLS showed higher diabetes prevalence and lower estimated glomerular filtration rate (eGFR) in females but not in males. The female in the reduced GLS group consumed more calcium channel blockers, beta-blockers, and renin-angiotensin blockers, while men consumed more diuretics. Age- and sex-related differences in the diastolic cardiac function are presented in Table 2. E/e’ was significantly lower in the reduced GLS group in females, but not in males, and the LA volume index was significantly greater in the reduced GLS group in females, but not in males (Supplementary Figure).

**Table 2:** Clinical characteristics and echocardiographic findings of study subjects by sex.

	Female				Male			
	Female total (n = 351)	Reduced GLS (< 18%) (n = 66)	Normal GLS ($\ge$ 18%) (n = 285)	*P*	Male total (n = 331)	Reduced GLS (< 18%) (n = 143)	Normal GLS ($\ge$ 18%) (n = 188)	*P*
Age, years	59.9 ± 12.3	68.0 ± 11.4	58.1 ± 11.8	< 0.001	56.3 ± 13.8	55.3 ± 13.8	57.0 ± 13.8	0.265
Age				< 0.001				0.770
$\le$ 66 years	237 (67.5)	26 (39.4)	211 (74.0)		245 (74.0)	107 (74.8)	138 (73.4)	
$>$ 66 years	114 (32.5)	40 (60.6)	74 (26.0)		86 (26.0)	36 (25.2)	50 (26.6)	
Body mass index, kg/m2	24.4 ± 2.90	24.6 ± 2.80	24.3 ± 2.92	0.412	24.8 ± 2.95	25.4 ± 3.25	24.3 ± 2.60	0.001
SBP	130.3 ± 18.4	142.3 ± 19.1	127.7 ± 17.2	< 0.001	130.8 ± 16.9	133.3 ± 19.1	128.3 ± 14.2	0.047
DBP	77.1 ± 11.5	84.6 ± 9.16	75.5 ± 11.3	< 0.001	81.3 ± 11.9	82.6 ± 12.9	80.0 ± 10.7	0.132
Alcohol	32 (9.1)	5 (7.6)	27 (9.5)	0.629	151 (45.6)	69 (48.3)	82 (43.6)	0.402
Cardiovascular risk factors								
Hypertension	166 (47.3)	45 (68.2)	121 (42.5)	< 0.001	173 (52.3)	81 (56.6)	92 (48.9)	0.164
Diabetes mellitus	46 (13.1)	15 (22.7)	31 (10.9)	0.010	60 (18.1)	27 (18.9)	33 (17.6)	0.756
Dyslipidemia	68 (19.4)	14 (21.2)	54 (18.9)	0.675	64 (19.3)	33 (23.1)	31 (16.5)	0.133
Obesity	139 (39.6)	31 (47.0)	108 (37.9)	0.174	145 (43.8)	74 (51.7)	71 (37.8)	0.011
Current smoking	7 (2.0)	3 (4.5)	4 (1.4)	0.100	112 (33.8)	55 (38.5)	57 (30.3)	0.121
Lab findings								
Glucose, mg/dL	106.1 ± 21.4	113.2. ± 28.8	104.5 ± 19.0	0.023	112.5 ± 35.1	113.8 ± 35.4	111.5 ± 34.9	0.562
Glycated hemoglobin, %	5.89 ± 0.67	6.15 ± 0.78	5.83 ± 0.63	0.002	5.90 ± 0.77	5.95 ± 0.91	5.86 ± 0.64	0.366
GFR_MDRD	91.6 ± 21.7	81.1 ± 22.2	94.1 ± 20.8	< 0.001	89.4 ± 20.7	91.5 ± 24.6	87.7 ± 17.0	0.101
Total cholesterol	187.2 ± 36.4	184.6 ± 38.4	187.8 ± 35.9	0.530	178.9 ± 38.9	181.4 ± 40.2	177.0 ± 37.8	0.306
LDL cholesterol	116.6 ± 34.1	113.3 ± 36.4	117.4 ± 33.6	0.430	111.9 ± 34.8	112.2 ± 36.5	111.6 ± 33.5	0.895
HDL cholesterol	53.8 ± 12.5	49.8 ± 11.6	54.8 ± 12.6	0.004	47.2 ± 12.1	47.0 ± 12.1	47.3 ± 12.2	0.847
Triglyceride	111.1 ± 59.8	119.2 ± 56.2	109.2 ± 60.5	0.220	126.6 ± 72.4	137.0 ± 80.1	118.7 ± 65.1	0.027
C-reactive protein	0.33 ± 1.17	0.70 ± 2.21	0.24 ± 0.65	0.152	0.42 ± 1.34	0.31 ± 0.84	0.50 ± 1.62	0.251
Concomitant medications								
Calcium channel blocker	96 (27.4)	25 (37.9)	71 (24.9)	0.033	115 (34.7)	53 (37.1)	62 (33.0)	0.440
Beta-blocker	56 (16.0)	17 (25.8)	39 (13.7)	0.016	50 (15.1)	24 (16.8)	26 (13.8)	0.457
Renin-angiotensin system blocker	93 (26.5)	27 (40.9)	66 (23.2)	0.003	93 (28.1)	50 (35.0)	43 (22.9)	0.015
Diuretic	21 (6.0)	6 (9.1)	15 (5.3)	0.237	13 (3.9)	10 (7.0)	3 (1.6)	0.012
Statin	142 (40.5)	34 (51.5)	108 (37.9)	0.042	136 (41.1)	61 (42.7)	75 (39.9)	0.613
Echocardiographic parameter								
GLS, %	19.8 ± 2.29	16.5 ± 1.30	20.6 ± 1.69	< 0.001	18.2 ± 2.10	16.3 ± 1.37	19.6 ± 1.29	< 0.001
LV ejection fraction, %	67.6 ± 4.42	66.1 ± 5.12	67.9 ± 4.17	0.008	66.4 ± 4.29	65.3 ± 4.34	67.2 ± 4.08	< 0.001
LV end-diastolic dimension, mm	47.1 ± 3.34	47.3 ± 4.04	47.0 ± 3.16	0.642	48.9 ± 3.39	49.2 ± 3.51	48.7 ± 3.30	0.192
LV end-systolic dimension, mm	29.3 ± 2.82	30.0 ± 3.34	29.2 ± 2.67	0.079	30.9 ± 3.05	31.5 ± 3.17	30.5 ± 2.89	0.002
LV septal wall thickness, mm	8.31 ± 1.18	8.79 ± 1.14	8.20 ± 1.16	< 0.001	9.08 ± 1.06	9.32 ± 1.06	8.90 ± 1.03	< 0.001
Posterior wall thickness, mm	8.29 ± 1.08	8.64 ± 1.04	8.21 ± 1.07	0.003	8.95 ± 1.10	9.25 ± 1.02	8.71 ± 1.11	< 0.001
LV mass index, g/m^2^	82.0 ± 18.0	89.8 ± 21.5	80.2 ± 16.6	< 0.001	85.5 ± 16.4	88.4 ± 17.7	83.2 ± 15.0	0.004
E wave velocity, cm/s	0.70 ± 0.32	0.72 ± 0.66	0.70 ± 0.15	0.811	0.62 ± 0.14	0.60 ± 0.15	0.64 ± 0.14	0.015
A wave velocity, cm/s	0.80 ± 0.51	0.99 ± 1.11	0.75 ± 0.19	0.084	0.68 ± 0.18	0.67 ± 0.17	0.69 ± 0.19	0.367
E/A	0.95 ± 0.35	0.77 ± 0.27	0.99 ± 0.35	< 0.001	0.97 ± 0.36	0.94 ± 0.33	0.99 ± 0.38	0.193
Deceleration time, ms	208.2 ± 46.4	216.0 ± 56.2	206.5 ± 43.9	0.208	210.5 ± 46.7	206.3 ± 47.7	216.7 ± 45.8	0.160
Septal e' velocity	7.22 ± 2.35	5.55 ± 1.82	7.61 ± 2.29	< 0.001	7.76 ± 4.58	7.07 ± 2.08	8.28 ± 5.74	0.017
E/e'	10.2 ± 3.19	12.4 ± 4.13	9.69 ± 2.69	< 0.001	8.61 ± 2.70	8.74 ± 2.57	8.52 ± 2.80	0.472
LA volume index, mL/m^2^	29.6 ± 8.72	33.5 ± 12.7	28.7 ± 7.25	0.004	27.3 ± 6.71	27.6 ± 6.63	27.2 ± 6.78	0.591
TR Vmax, m/s	2.26 ± 0.26	2.27 ± 0.27	2.26 ± 0.26	0.720	2.21 ± 0.27	2.20 ± 0.29	2.22 ± 0.26	0.659

GLS, global longitudinal strain.

Numbers are mean ± standard deviation or n (%). GLS, global longitudinal strain.

HTN : previous diagnosis, current antihypertensive medications or SBP and/or DB*P* >  = 140/90.

DM : previous diagnosis, current anti-diabetic medications or fasting blood glucose level >  = 126 mg/dL.

DL : previous diagnosis, current use of anti-dyslipidemic medications or LDL >  = 160 mg/dL

A Scatter plot was used to evaluate the correlation between age and GLS, and to compare the differences between males and females. GLS decreased with age in females; however, there was no significant correlation between age and GLS in males (Fig. 1).

**Figure 2:**
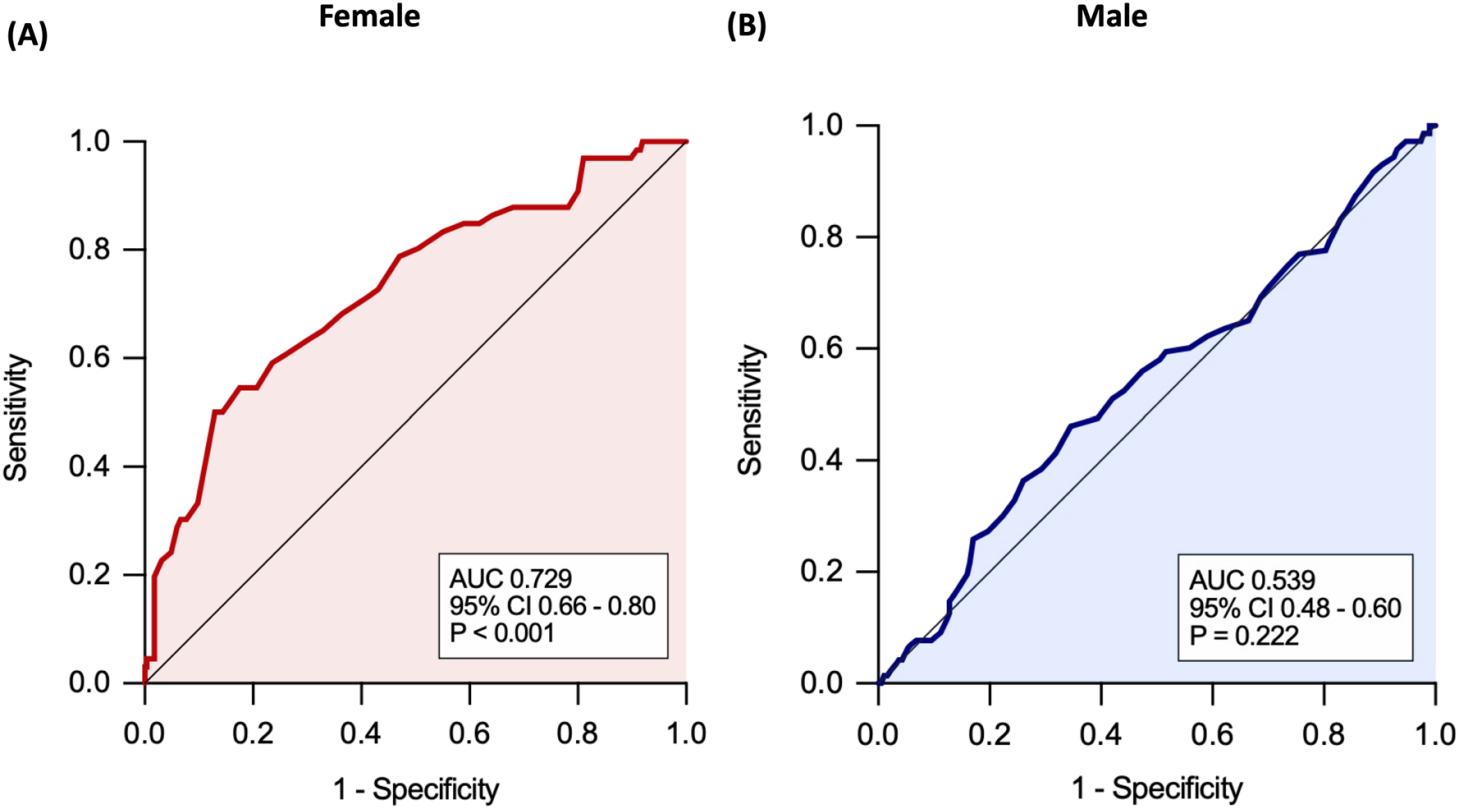
Receiver operating characteristic curve of GLS for predicting age in both sexes. The area under the ROC curve was 0.729 in females and 0.539 in males.

**Figure 3:**
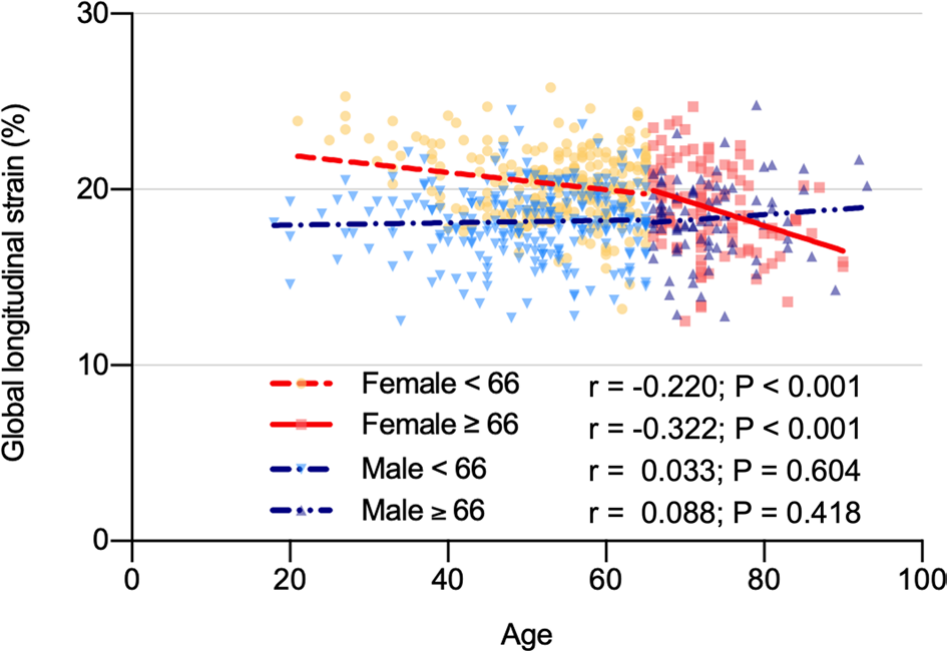
Correlation between age and GLS in age groups by sex (females, age less than 66 years, r = 0.220, *P* < 0.001; age over 66 years, r = 0.322, *P* < 0.001).

Owing to age being statistically higher in participants with reduced GLS, ROC curve analysis was performed to compare the significance of age between sexes. The area under the ROC curve of reduced GLS in females was 0.729 (95% CI 0.66–0.80, *P* < 0.001, Fig. 2A). The area under the ROC curve of reduced GLS in males was 0.539 (95% CI 0.48–0.60, *P* = 0.222; Fig. 2B). The optimal cutoff age for predicting the risk of reduced GLS in females was 66.5 years. The sex-specific differences in GLS by age 66 years are shown in Fig. 3. At the age of 66 years, the difference in slope was evident in the scatter plot of age and GLS in females, but not in males.

### Univariate and multivariate analysis showing clinical factors associated with reduced GLS compared by sex

Univariate and multivariate analyses were conducted with variables that showed significant differences between the two groups of females (Table 3). Hypertension (OR 3.69; 95% CI 1.52‒8.97; *P* = 0.004) and low GFR_MDRD (Modification of Diet in Renal Disease) (OR 0.97; 95% CI 0.95–0.99; *P* = 0.006) were associated with reduced GLS, and age over 66 years was independently associated with reduced GLS (OR 2.66; 95% CI 1.22–5.76; *P* = 0.014) in females.

**Table 3:** Comparison between univariate model and multivariate model.

Characteristic	Risk factors for reduced GLS							
	Female				Male			
	Univariate		Multivariate		Univariate		Multivariate	
	OR (95% CI)	*P*	OR (95% CI)	*P*	OR (95% CI)	*P*	OR (95% CI)	*P*
Age $>$ 66 years	3.53 (1.68–7.43)	0.001	2.66 (1.22–5.76)	0.014	0.93 (0.57–1.53)	0.770	1.02 (0.60–1.74)	0.952
Hypertension	2.90 (1.65–5.13)	< 0.001	3.69 (1.52–8.97)	0.004	1.36 (0.88–2.11)	0.165	1.03 (0.62–1.73)	0.902
Diabetes mellitus	2.41 (1.21–4.79)	0.012	1.16 (0.41–3.27)	0.777	1.09 (0.62–1.92)	0.756	0.99 (0.55–1.81)	0.983
BMI, kg/m2					1.14 (1.06–1.24)	0.001	1.13 (1.00–1.29)	0.057
Obesity (BMI $\ge 25)$					1.77 (1.14–2.75)	0.011	0.88 (0.42–1.82)	0.726
GFR_MDRD	0.97 (0.95–0.98)	< 0.001	0.97 (0.95–0.99)	0.006				
HDL cholesterol	1.62 (0.94–2.77)	0.081	0.99 (0.96–1.03)	0.703				
Triglyceride					1.00 (1.00–1.01)	0.026	1.00 (1.00–1.01)	0.151
C-reactive protein	1.36 (0.98–1.89)	0.065	1.31 (0.90–1.90)	0.159				
Calcium channel blocker	1.84 (1.04–3.23)	0.035	0.69 (0.27–1.78)	0.440				
Beta-blocker	2.19 (1.15–4.18)	0.018	1.13 (0.46–2.77)	0.786				
RAAS blocker	2.30 (1.31–4.03)	0.004	0.69 (0.27–1.78)	0.442	1.81 (1.12–2.94)	0.016	1.46 (0.83–2.56)	0.192
Statin	1.74 (1.02–2.99)	0.044	1.17 (0.50–2.73)	0.722				

GLS, global longitudinal strain.

There were some statistically significant variables in the differences between reduced and normal GLS groups in males; however, these variables were not associated with reduced GLS in the multivariate analysis.

## Discussions

In this study, we investigated the pattern of GLS change in patients with normal LVEF and the correlation between GLS and age according to sex. Although mean GLS was higher in females than in males (19.8% ± 2.29% vs. 18.2% ± 2.10%, respectively), GLS decreased significantly with aging only in females, and the cut-off age was 66 years. Therefore, GLS might help to assess longitudinal dysfunction with normal LVEF in older females. Moreover, this sex-specific reduced GLS may be a potential mechanisms for higher prevalence of HFpEF in older females. To the best of our knowledge, this is the first study to show a relationship between GLS and age and to offer the cut-off age value for reduced GLS in females with normal LVEF.

### Normal LVEF and reduced GLS.

LVEF is widely used as a prognostic marker and has been a standard for classifying HF, but its use is limited in patients with HFpEF whose LVEF is preserved. GLS may be a method used in this setting. Previous studies suggested that GLS is reduced before LVEF is impaired^9^; therefore, it has been proposed as an important parameter of early-stage or subclinical LV dysfunction^10,11^.

A prospective study demonstrated that reduced GLS was a strong predictor of HF admission among patients in stage A (asymptomatic at risk for heart failure) and B (asymptomatic with left ventricular dysfunction) HF. And patients in stage B HF with reduced GLS were significantly associated with a high risk of developing overt HF^19^. Moreover, GLS is a superior predictor of cardiac events and mortality than LVEF in early cardiomyopathies^20–22^. Recently, decreased longitudinal systolic function has been suggested to be an important mechanism in HFpEF^4^ and a meta-analysis reported significantly lower LV GLS in patients with HFpEF^12^. In this respect, LV GLS is expected to be helpful as a diagnostic method for HFpEF patients with a normal LVEF.

### Previous studies of differences in GLS according to age and sex

Previous studies have reported differences in GLS according to advanced age and sex. In the Framingham study, echocardiographic measurements of LV myocardial strain were conducted in healthy adults (mean age, 63 years; 64% females)^13^. This study demonstrated that reduced values of GLS were associated with advanced age, and females showed greater GLS than males^13^. In the ARIC (Atherosclerosis Risk in Communities) study, LV mechanics in participants without prevalent heart failure (mean age 76 years, 61% females) were evaluated, and this study reported that advanced age was associated with reduced GLS and female showed greater GLS^6^. In our study (mean age, 58 years; 51% females), however, there was no significant difference in age between participants with reduced and normal GLS, but mean GLS was higher in females than in males (19.8% ± 2.29% vs. 18.2% ± 2.10%, respectively) before considering age. These differences GLS by age might be owing to the different ages of the study participants and the proportion of females. In our study, although mean GLS value was greater in females than males, it significantly decreased with age only in females. Taken together, based on our results, a higher proportion of older females among the study participants might have affected the association between advanced age and GLS.

Meanwhile, Park et al. determined the reference values for LV longitudinal strain using information from normal Korean patients (mean age, 47 years; 52% females)^14^. In this study, there were no significant differences in GLS between the different age groups, but females had higher LV GLS values than males^14^, which is in line with our results. However, with respect to age, the results were different when comparing LV GLS between age groups (< 40 years vs. > 60 years) in both sexes; younger females showed significantly higher GLS values than younger males, but in the older group, no significant differences were observed in this study. These results are somewhat similar with those of our study that there was no significant difference in age between participants with reduced GLS and normal GLS, and mean GLS was higher in females than in males (19.8% ± 2.29% vs. 18.2% ± 2.10%, respectively) before considering age. However, unlike the study by Park et al., our study demonstrated that females aged over 66 years showed significantly lower GLS. These different results might be owing to the different mean ages of the study participants; mean age in Park et al.’s study was 47 years, which implies that enrolled participants older than 66 years might not be enough to estimate the association between sex and GLS by age.

Based on these previous reports, considering age cut-off to interpret the GLS values differently by sex is significant and from this point of view, our results that older females showed significantly lower GLS than males, and that the cut-off age for predicting the risk of reduced GLS in female was 66 years, may deserve attention.

### Why GLS decreases with age in female

Longitudinal function decreases with aging; therefore, myocardial twist may increase to preserve ejection fraction^6,23^. Compared to the myocardium of males, that of females exhibit lower myocardial turnover, apoptosis, fibrotic changes, LV volumes, and better contractile reserve^5,6,24^. To deliver sufficient cardiac output and satisfy tissue metabolic demand, better systolic function and more torsion may be necessary due to the significantly lower LV volume in females^6^. These differences could be explained by differences in sex hormones, such as estrogen, which may be advantageous in slowing the course of heart diseases^5^. Functional estrogen receptors (ERs [α and β]) were found in the ventricular myocardium, and estrogen binding has both genomic and non-genomic consequences^25^. Novotny et al. studied the protective effect of ERα in aged female rat hearts and found that ERα activation was effective in lowering necrotic and/or apoptotic cell death as well as ischemia–reperfusion damage in the aged heart^26^. Therefore, menopause is considered to have a relationship with reduced cardiac function and adverse LV remodeling. Several studies have shown that menopause adversely affects the myocardial performance index and velocities and Doppler aortic flow indices of LV systolic function^27,28^, thus estrogen level changes diminished LV systolic and diastolic functions. Maluleke et al. studied female rats, performed ovariectomy to cause menopause in half of rats, and compared cardiac function by conducting echocardiography in two rat groups. This study showed that ovariectomized rats had significantly decreased GLS but showed no significant difference after adjusting for BP and body mass. A lack of estrogen does not result in LV dysfunction, impaired myocardial deformation, or cardiac remodeling^29^. Our study also showed that age and hypertension were independent factors for reduced GLS in multivariable analysis. Taken together, the mechanisms of decreasing GLS in older females are not clearly understood; reduced cardioprotective effects such as estrogen fall in older females may cause GLS to decline, and traditional cerebrovascular diseases such as hypertension accompanied by menopause might cause cardiac dysfunction, which shows reduced GLS.

### Clinical implications

HFpEF is more frequent in old age and females^30,31^ and is significantly associated with cardiovascular morbidity and mortality^32^. Therefore, it is essential to detect HFpEF early in older females and provide proper management. Our results demonstrated that GLS decreased with increasing age in females, and decreased GLS values were remarkable in females aged 66 years or older. This suggests that measuring LV GLS in older females, especially in those aged over 66 years, may provide information on LV dysfunction and allow clinicians to manage it early and adequately. To generalize our findings, further studies with larger populations are warranted.

### Study limitations

This study has several limitations. First, this study included only Korean participants; therefore, it would not be valid to generalize the results to other populations. Second, this study found that increasing age in females is associated with decreasing GLS, but it could not analyze factors related to GLS due to lack of data on female factors, including menopause duration or hormone therapies that may have played a role. Third, this study was cross-sectional; therefore cardiovascular outcomes could not be compared between sexes in participants with reduced GLS and normal LVEF. Forth, due to the lack of information on NT-proBNP or heart failure symptoms, we were unable to analyze the association between reduced GLS and HFpEF in our study population. Finally, although we adjusted for several confounders and conducted a multivariate analysis, there could be possible unrecognized confounders that may have played a role in the observed associations.

## Conclusions

In participants with normal LVEF, GLS decreased with increasing age in females, but not in males. In particular, the risk of reduced GLS was highly significant in females aged over 66 years. Our findings suggest that the assessment of LV function by GLS could be useful in adults with normal LVEF, especially in older females.

### Supplementary Information

Supplementary Information.

## Data Availability

All data generated or analyzed during this study are included in the article.
