# Supplementary material for: Sex difference in the age-related decline of global longitudinal strain of left ventricle
Source: Sci Rep. 2023 Oct 27;13:18441. doi: 10.1038/s41598-023-42286-9 (PMC10611699; doi:10.1038/s41598-023-42286-9)

**Sex difference in the age-related decline of global longitudinal strain of left ventricle**

Kyung-Yeon Lee^1^*, Hack-Lyoung Kim^1^*, Kyung-Jin Kim^2^

^1^Division of Cardiology, Department of Internal Medicine, Boramae Medical Center, Seoul National University College of Medicine, Seoul, Korea.

^2^Department of Internal Medicine, Ewha Womans University School of Medicine, Seoul, Korea.

**Supplementary Materials**

**Table**

**Supplementary Table S1. Clinical characteristics and echocardiographic findings of study subjects.**

**Supplementary Figure**

**Supplementary Figure 1. Age- and sex-related differences in cardiac dimensions and diastolic index between age groups (Left ventricular mass index, LVMI, panel A; left atrial volume index, LAVI, panel B; E/A, panel C; E/E’, panel D).**

Supplementary Table S1. Clinical characteristics and echocardiographic findings of study subjects.

|  | **Total**  **(n= 682)** | **Reduced GLS**  **(<18%)**  **(n = 209)** | **Normal GLS**  **(**$\boldsymbol{\geq}$**18%)**  **(n = 473)** | **P** |
| --- | --- | --- | --- | --- |
| **Age, years** | 58.2 ± 13.2 | 59.3 ± 14.4 | 57.6 ± 12.6 | 0.128 |
| **Men, sex** | 331 (48.5) | 143 (68.4) | 188 (39.7) | <0.001 |
| **Women, sex** | 351 (51.5) | 66 (31.6) | 285 (60.3) | <0.001 |
| **Body mass index, kg/m2** | 24.5 ± 2.93 | 25.2 ± 3.13 | 24.3 ± 2.80 | <0.001 |
| **SBP** | 130.5 ± 17.7 | 135.9 ± 19.4 | 127.9 ± 16.2 | <0.001 |
| **DBP** | 79.1 ± 11.9 | 83.2 ± 11.9 | 77.1 ± 11.3 | <0.001 |
| **HR** | 67.8 ± 10.7 | 69.9 ± 11.9 | 66.8 ± 10.0 | 0.007 |
| **Alcohol** | 183 (26.8) | 74 (35.4) | 109 (23.0) | 0.001 |
| **Cardiovascular risk factors** |  |  |  |  |
| **Hypertension** | 339 (49.7) | 126 (60.3) | 213 (45.0) | <0.001 |
| **Diabetes mellitus** | 106 (15.5) | 42 (20.1) | 64 (13.5) | 0.029 |
| **Dyslipidemia** | 132 (19.4) | 47 (22.5) | 85 (18.0) | 0.169 |
| **Obesity** | 284 (41.6) | 105 (50.2) | 179 (37.8) | 0.002 |
| **Current smoking** | 119 (17.4) | 58 (27.8) | 61 (12.9) | <0.001 |
| **Lab findings** |  |  |  |  |
| **Glucose, mg/dL** | 109.2 ± 29.0 | 113.6 ± 33.4 | 107.3 ± 26.6 | 0.009 |
| **Glycated hemoglobin, %** | 5.90 ± 0.72 | 6.01 ± 0.88 | 5.84 ± 0.63 | 0.012 |
| **GFR_MDRD** | 90.5 ± 21.2 | 88.2 ± 24.3 | 91.5 ± 19.6 | 0.059 |
| **Total cholesterol** | 183.2 ± 37.8 | 182.4 ± 39.6 | 183.5 ± 37.0 | 0.734 |
| **LDL cholesterol** | 114.4 ± 34.5 | 112.6 ± 36.4 | 115.2 ± 33.7 | 0.399 |
| **HDL cholesterol** | 50.6 ± 12.8 | 47.9 ± 12.0 | 51.8 ± 12.9 | <0.001 |
| **Triglyceride** | 118.6 ± 66.6 | 131.4 ± 73.7 | 113.0 ± 62.5 | 0.001 |
| **C-reactive protein** | 0.38 ± 1.26 | 0.44 ± 1.43 | 0.35 ± 1.17 | 0.468 |
| **Concomitant medications** |  |  |  |  |
| **Calcium channel blocker** | 211 (30.9) | 78 (37.3) | 133 (28.1) | 0.017 |
| **Beta-blocker** | 106 (15.5) | 41 (19.6) | 65 (13.7) | 0.051 |
| **Renin-angiotensin system blocker** | 186 (27.3) | 77 (36.8) | 109 (23.0) | <0.001 |
| **Diuretic** | 34 (5.0) | 16 (7.7) | 18 (3.8) | 0.033 |
| **Statin** | 278 (40.8) | 95 (45.5) | 183 (38.7) | 0.097 |
| **Echocardiographic parameter** | | | | |
| **GLS, %** | 19.1 ± 2.35 | 16.4 ± 1.35 | 20.2 ± 1.62 | <0.001 |
| **LV ejection fraction, %** | 67.0 ± 4.39 | 65.6 ± 4.60 | 67.6 ± 4.14 | <0.001 |
| **LV end-diastolic dimension, mm** | 48.0 ± 3.48 | 48.6 ± 3.78 | 47.7 ± 3.31 | 0.004 |
| **LV end-systolic dimension, mm** | 30.1 ± 3.04 | 31.0 ± 3.30 | 29.7 ± 2.82 | <0.001 |
| **LV septal wall thickness, mm** | 8.68 ± 1.19 | 9.15 ± 1.11 | 8.48 ± 1.16 | <0.001 |
| **Posterior wall thickness, mm** | 8.61 ± 1.14 | 9.06 ± 1.06 | 8.41 ± 1.11 | <0.001 |
| **LV mass index, g/ m²** | 83.7 ± 17.3 | 88.9 ± 18.9 | 81.4 ± 16.0 | <0.001 |
| **E wave velocity, cm/s** | 0.66 ± 0.25 | 0.64 ± 0.39 | 0.67 ± 0.15 | 0.67 |
| **A wave velocity, cm/s** | 0.74 ± 0.39 | 0.77 ± 0.65 | 0.73 ± 0.19 | 0.157 |
| **E/A** | 0.96 ± 0.35 | 0.89 ± 0.32 | 0.99 ± 0.36 | <0.001 |
| **Deceleration time, ms** | 209.3 ± 46.6 | 209.3 ± 50.6 | 209.3 ± 44.8 | 0.996 |
| **Septal e' velocity** | 7.48 ± 3.61 | 6.59 ± 2.12 | 7.88 ± 4.04 | <0.001 |
| **E/e'** | 9.43 ± 3.07 | 9.90 ± 3.58 | 9.23 ± 2.79 | 0.016 |
| **LA volume index, mL/ m²** | 28.5 ± 7.89 | 29.4 ± 9.37 | 28.1 ± 7.10 | 0.042 |
| **TR Vmax, m/s** | 2.24 ± 0.26 | 2.23 ± 0.28 | 2.25 ± 0.26 | 0.513 |

Numbers are mean ± standard deviation or n (%). GLS, global longitudinal strain

HTN : previous diagnosis, current antihypertensive medications or SBP and/or DBP ≥140/90

DM : previous diagnosis, current anti-diabetic medications or fasting blood glucose level ≥ 126mg/dL

DL : previous diagnosis, current use of anti-dyslipidemic medications or LDL ≥ 160mg/d

**Supplementary Figure 1.** Age- and sex-related differences in cardiac dimensions and diastolic index between age groups (Left ventricular mass index, LVMI, panel A; left atrial volume index, LAVI, panel B; E/A, panel C; E/E’, panel D)


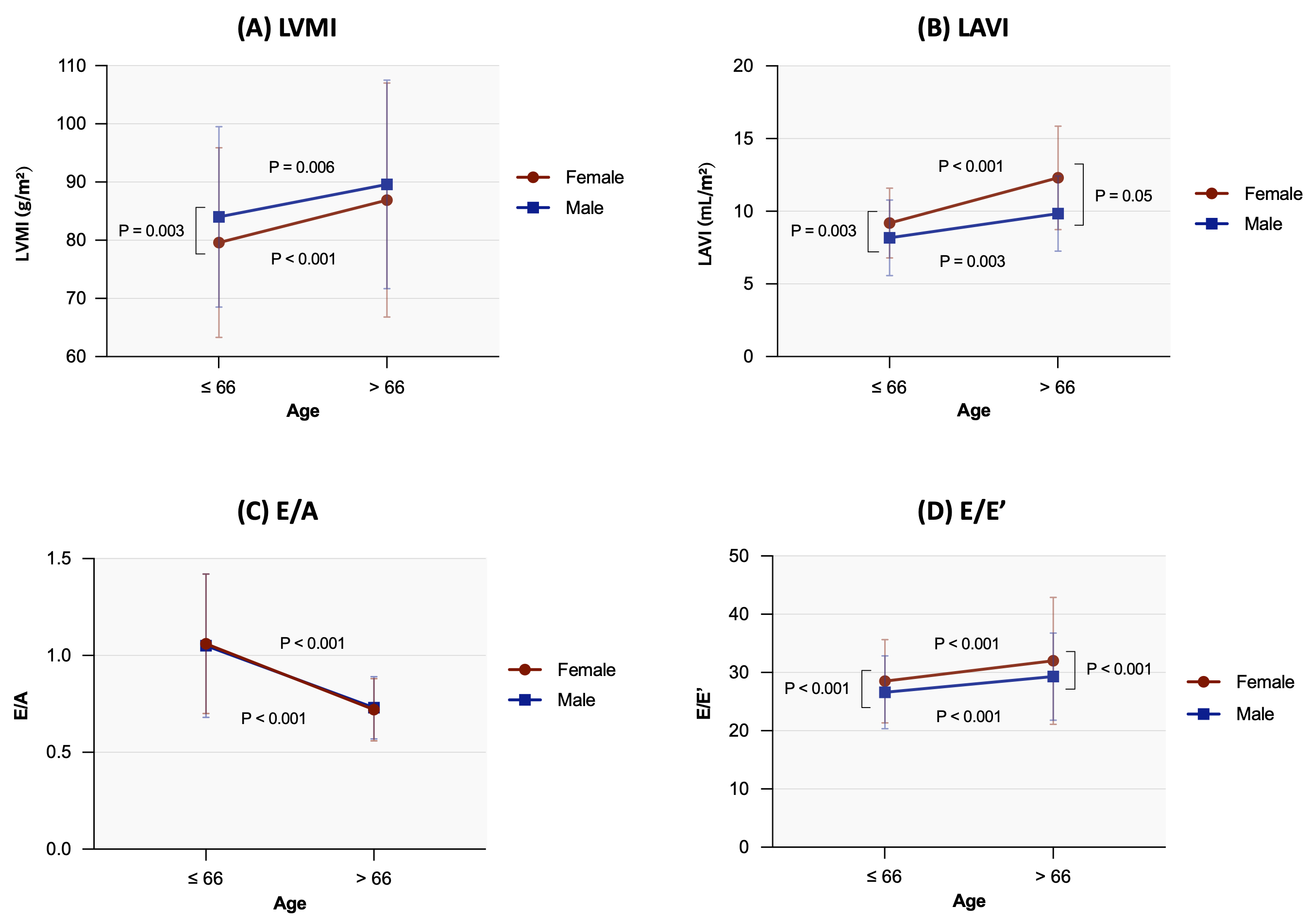

Supplement: Supplementary file 1 — Supplementary Information. [file 41598_2023_42286_MOESM1_ESM.docx]
